# Anomaly Detection Trusted Hardware Sensors for Critical Infrastructure Legacy Devices

**DOI:** 10.3390/s20113092

**Published:** 2020-05-30

**Authors:** Apostolos P. Fournaris, Charis Dimopoulos, Konstantinos Lampropoulos, Odysseas Koufopavlou

**Affiliations:** 1 Industrial Systems Institute, R.C. ATHENA, Patras Science Park, 26504 Platani-Patras, Greece; 2 Electrical and Computer Engineering Department, University of Patras, Rion Campus, 26504 Rion-Patras, Greece; c.dimopoulos@upnet.gr (C.D.); klamprop@ece.upatras.gr (K.L.); odysseas@ece.upatras.gr (O.K.)

**Keywords:** security, hardware design, trust, cryptography, anomaly detection

## Abstract

Critical infrastructures and associated real time Informational systems need some security protection mechanisms that will be able to detect and respond to possible attacks. For this reason, Anomaly Detection Systems (ADS), as part of a Security Information and Event Management (SIEM) system, are needed for constantly monitoring and identifying potential threats inside an Information Technology (IT) system. Typically, ADS collect information from various sources within a CI system using security sensors or agents and correlate that information so as to identify anomaly events. Such sensors though in a CI setting (factories, power plants, remote locations) may be placed in open areas and left unattended, thus becoming targets themselves of security attacks. They can be tampering and malicious manipulated so that they provide false data that may lead an ADS or SIEM system to falsely comprehend the CI current security status. In this paper, we describe existing approaches on security monitoring in critical infrastructures and focus on how to collect security sensor–agent information in a secure and trusted way. We then introduce the concept of hardware assisted security sensor information collection that improves the level of trust (by hardware means) and also increases the responsiveness of the sensor. Thus, we propose a Hardware Security Token (HST) that when connected to a CI host, it acts as a secure anchor for security agent information collection. We describe the HST functionality, its association with a host device, its expected role and its log monitoring mechanism. We also provide information on how security can be established between the host device and the HST. Then, we introduce and describe the necessary host components that need to be established in order to guarantee a high security level and correct HST functionality. We also provide a realization–implementation of the HST overall concept in a FPGA SoC evaluation board and describe how the HST implementation can be controlled. In addition, in the paper, two case studies where the HST has been used in practice and its functionality have been validated (one case study on a real critical infrastructure test site and another where a critical industrial infrastructure was emulated in our lab) are described. Finally, results taken from these two case studies are presented, showing actual measurements for the in-field HST usage.

## 1. Introduction

In recent years, many critical infrastructures (CIs) around the world adopted various Information and Communication Technologies (ICT) advances, in an effort to become more flexible and cost effective. However, this adaptation was not made carefully and with a thorough evaluation on the implications it introduced to their security. Numerous new devices with advanced computation power and connectivity capabilities are constantly been installed in CIs and the—once closed and isolated CI systems—are becoming more and more vulnerable to new types of threats and attacks. Various reports and research teams have proved how dangerous this situation is. For example, a team called “SCADA StrangeLove team” was able, back in 2013, to get full control of various industrial infrastructures (energy, oil and gas, chemical and transportation CIs). They claimed to have found more than 60,000 online control systems that were exposed. Furthermore, even though nowadays CIs are more secure, the number and sophistication of cyberattacks is still increasing [1]. There is a critical need to fortify CIs to the maximum possible, since a major cyberattack in one of them may cause severe problems not only at a technical level but also in the economy, public safety, etc. [2].

On the other hand, protecting CI systems is a highly complicated task. A large number of diverse security systems and protection mechanisms must collaborate [3,4]. Solutions like Anomaly Detection Systems (ADS), Intrusion Detection (IDS), Antivirus tools for Malwares and Ransomwares, DDoS protection, Endpoint security, Hardware, protection for CI devices, Access control, etc., are just few of the technical tools that can be used to fortify such complex environments [5]. In addition, apart from the technical parts, a CI security must also include human training (personnel, users, etc.), Private-Public Partnerships, Assessments, Vulnerability Analyses, etc.

Usually the above security tools and solutions are integrated inside a Unified Threat Management (UTM) system or a Security Information and Event Management (SIEM) system. The difference between a SIEM and a UTM is that the SIEM does not exactly integrate security components but only collects reporting information (e.g., logs, reports, events, etc.) and combines it with input from other sources in order to “assemble a puzzle” which would eventually identify a possible security risk.

Inside this wide area of security solutions, this work examines innovations on a very specific aspect of CI protection—the design of trusted sensors for Anomaly Detection Systems (ADS) and SIEMs. An ADS can be described as a solution which extends the functionality of an Intrusion Detection System (IDS). In particular, the ADS not only monitors very specific, predetermined network metrics but also collects information from multiple other sources to estimate the security status of an IT system. Such sources can be distributed sensors inside a CI that generate logs which are collected by a centralized ADS or SIEM analyzer. The success rate of an ADS detection process (small false positives or negatives number) heavily relies on the quality ADS analyzer algorithm (Machine Learning techniques are also used nowadays) and the accuracy of the collected data [6,7]. Obviously, data from maliciously manipulated sensors can lead an ADS in producing false results and keep CI administration ignorant or falsely alert on a possible cybersecurity attack [8,9].

In this paper, we review the option of using hardware means in order to secure sensors’ ADS/SIEM transmitted data, instilling trust in the overall process. In addition, extending the work in [2], we propose a Hardware Security Token to be physically connected to legacy CI devices and act as a trusted ADS sensor for failed access attempts as well as a mechanism for providing authentication and integrity to sensor’s collected data. In the paper, we analyze the HST architecture and approach and we describe how it can achieve a level of trust in the associated host device using an appropriate security protocol. We also describe the main HST functionality achievable through the use of a dedicated host software program for accessing the HST as well as the HST log reporting mechanism on the ADS monitoring system. Then we describe a realization–implementation of the HST using an FPGA SoC evaluation board and we show how the HST services can be accessed. Finally, in the paper, two case studies using the HST in practice are described (one case study on a real critical infrastructure test site and another where a critical industrial infrastructure was emulated in our lab) and results are presented showing the HST capabilities in practice. The rest of the paper is organized as follows. In Section 2, an overview of a CI ADS sensors is made, security issues that may arise are described and a relevant threat model is presented. In Section 3, mechanisms to create trusted ADS sensors are described and in Section 4 the hardware assisted sensor approach, architecture, and functionality are proposed. Section 5 provides a realization of the HST along with use case scenarios of its usage and Section 6 concludes the paper.

## 2. Critical Infrastructure Security Monitoring System Anomaly Detection Sensors

Considering the security threats and challenges that many critical infrastructures have, there is a considerable need to continuously monitor such infrastructures during their regular operation for security anomalies that can be linked to some security attack [10,11,12,13]. Typical IT systems have a series of well-developed tools that, using a wide range of technologies and methods, can detect, respond and mitigate security attacks. The generic category of run-time monitoring systems may comprise of various components like intrusion detection systems (IDS), zero-vulnerability malware detectors and anomaly detectors that are all interconnected under a Security Information and Event Management (SIEM) system [7,14]. The Runtime monitoring Anomaly Detection mechanism is usually responsible for the correlation between various events and logs to extract security alerts and make attack mitigation suggestions. However, CI runtime security monitoring must consider the CI specificities that differ from those of a typical IT system [15,16].

CI systems (CIS) have a close association with the physical world (they monitor and respond to physical processes), thus they constitute an ideal realization of cyberphysical systems (or system of systems) and should be approached in that way in terms of security. According to [6], there are four basic characteristics that distinguish a CIS from typical IT systems in terms of runtime security intrusion detection. Due to their connection between the cyber and the physical world, the CIS devices measure physical phenomena and perform physical processes that are governed by the laws of physics. Thus, a CIS security monitoring system must perform physical process monitoring. Furthermore, typical CIS use many OT components, thus they are highly focused on automation and time driven processes that realize closed control loops operating with no human intervention (and its associated unpredictability). This kind of behavior focuses on Machine to Machine communications, increases the regularity and predictability of the CIS activities and makes them attractive to attackers [3]. Thus, the CIS security monitoring system should be able to monitor regularly closed control loops. Thirdly, the attack surface of a CIS is considerably broader than that of an IT system. CISs consist of many heterogeneous subsystems, including IT and OT devices. They follow a broad range of different, not IT related, control protocols like ISA 100, Modbus, CAN, etc. Some of these devices and protocols have proprietary software or standards that may make IT countermeasures unfitting [2,5]. This reason along with the fact that a successful CIS attack has high impact and thus high payoff, attracts very skilled attackers that can mount very sophisticated attacks on CPSs and CIS [13]. Such attacks are usually very hard to discover and document. Attackers exploit CIS zero-day vulnerabilities which would render many IT security monitoring toolsets useless (e.g., knowledge-based toolsets [6]).

Lastly, many CIS consist of legacy hardware that is difficult to modify or physically access. Such components may be partially analog, have very limited installed software resources and can be dictated by physical processes. The biggest challenge in such legacy devices is how to install security monitoring sensors on them and how to predict/model their behavior correctly in order to detect possible anomalies. Since legacy devices do not have many computational resources, it becomes hard for the monitoring system to retain its real-time responsiveness when collecting security metrics from them.

Runtime Security monitoring in the CIS domain, considering the above specificities, can take various forms. Monitoring relies on two core functions—the collection of data from various CIS sources and the analysis of data in a dedicated runtime security monitoring subsystem. To achieve appropriate data collection, the security monitoring system must deploy security agent software on the monitored CIS devices or introduce virtual entities (Virtual Machines) for data collection within the CIS infrastructure. All collected data are analyzed in the CIS runtime security monitoring system that uses data mining, machine learning, pattern recognition or statistical data analysis to extract metrics on security issues that may take place inside the CIS at runtime. Such issues may be possible incidents, threats that can be binary characterized as bad/good or continuously characterized by a specific significance grade. The performance of the security monitoring system is measured by the False Positive Rate (FPR), the False Negative Rate (FNR) and the True Positive Rate (TPR). The system is also measured in terms of incident detection latency and consumed resources number, computational overhead, excessive network traffic and power consumption [6].

The functionality and services SIEM and ADS applications provide can be considered a necessity and an integral component of a CIS security monitoring system. The basis of their functionality lies in the collection of various metrics reporting the health of a computing system and its network done by a broad network of local and remote sensor entities. These programs vary from honeypots, package analyzers, port scanner to antivirus or antimalware solutions. As a valuable source of ADS input data, one can consider the various OS activities, such as successful or failed authentication and authorization, collected by the OS’ system log manager. The purpose of the above is for those sensors to track specific activities, log them and provide the necessary information to the ADS when some prerequisites defined by the security administrator are met. The collection of those events can be in real time or near real time, aiding the security administrators with visual cues in their attempt to monitor the overall system’s security status and informing them of abnormalities that may lead to a compromised system. ADS sensors typically can be deployed as a software program installed on a host machine.

The heterogeneity CIS exhibit is directly reflected in the diverse nature of the sensors necessary to collect and log information for a CIS ADS. This variety of sensors, coupled with the critical nature of the overall system and the exposure various CI devices have on the CI premises (remote locations, power plants, factories) greatly increases the risk of successful device tampering or manipulation by an adversary. This type of compromise (often executed on the hardware level) is mostly ignored by the device’s software ADS sensor and often leads to data manipulation of the ADS logging mechanism. As an implication, the ADS is provided with fabricated data, leading to either the suppression of real or creation of false anomaly events on the security monitoring mechanism.

### 2.1. Threat Model

There exist only a few works that attempt to ensure trust in the information collection mechanism from a network’s end points of a CIS security monitoring and ADS system [17,18,19]. Some approaches rely on securing the communication channel between end device and ADS/IDS monitoring system [18] or rely on securing the end node itself by ad-hoc, trusted computing based, mechanisms [17,20]. Without loss of generality, we can assume that the main threats on the end point security monitoring sensors can be associated with attacks on a CIS end node that disrupt the sensor logging mechanism. This disruption can be achieved by manipulation of the communication channel between the device’s sensor and the remote ADS/security monitor leading to integrity or authenticity threats or can be achieved by hijacking the CIS node itself. In the second case, our threat model also considers that it is realistic for an attacker to gain access to the CIS nodes physical storage disk and not have full control of the node’s memory (the attacker does not have root access to the CIS node’s operating system). We also consider in our threat model threats related to physical attacks using some invasive (tampering), semi invasive (fault injection) or non invasive attack (side channel analysis), since we can assume that CIS end nodes may be left unattended in hostile environments [21,22]. We also consider failed identification and authentication and failed access attempts on the node or the sensor itself as active threats. Such failures should be logged and sent in a trusted/secure way to the anomaly detection security monitor system for analysis.

## 3. Introducing Trust on Software Sensors

In cases when there is a need to instill trust on a computing system, a very efficient method involves the introduction of a trusted computing base (TCB). This level of trust is usually achieved through the inclusion of a hardware component (a security token) in the device’s architecture. Through this secure environment, the TCB is able to act as a point of reference for the overall system, acting as a root of trust. Apart from the security critical operations, this secure computation environment (TCB) can also be considered hard to tamper with [23]. Both industry and academia have proposed several approaches for the most efficient way this hardware root of trust mechanism should be realized. One of the most important approaches is the specifications provided by Trusted Computing Group (TCG), which aims to instill trust on a system by guarding critical data (private keys), by blocking the execution of potentially harmful code and by attesting the system’s trust level to other entities. Even from boot time, the system’s security status is monitored continuously in order to achieve this level of trust, usually further enhanced by hardware mechanisms included in the TCB. Since software solutions do not provide adequate protection individually, TCG specifies a Hardware Security Module called the Trusted Platform Module (TPM), capable of acting as trust anchor within a computer system [24,25].

### 3.1. Using Trusted Platform Modules

For a CI device to be considered trusted, the inclusion of a TPM chip is of paramount importance, coupled with the necessary software stack embedded in the OS kernel in order to support the TCG trusted computing functionality. As a result, a TCG Trusted computing enabled device is capable of creating a secure environment to execute an ADS sensor’s software code [17]. Despite the broad adoption of TPMs in Personal Computer use, they are not yet utilized in the CI domain and cyberphysical systems. This issue is also present in the majority of embedded devices or CI control elements (e.g., Programmable Logic Controllers—PLCs). For these types of devices without a TPM and constrained in resources and power consumption, TCG’s suggestion is the use of the Device Identifier Composition Engine (DICE) mechanism, which nonetheless is far from adopted in CI system OT and IT end nodes that are not PCs.

### 3.2. Using Virtual Environments

An additional approach that can be used to instill trust in a CI device is Hardware virtualization. The general concept is the creation of isolated execution environments that under certain conditions can be considered trusted. Using this technique, critical or sensitive applications and their accompanying data can be directed towards these trusted areas of virtual machines (VMs) running on virtualized hardware. By expanding this logic, an isolated OS has the ability to operate on such a VM. If access to this VM is under the control of a Trusted Computing Base (TCB) program on the CPU, the OS can be considered secure and isolated from any other untrusted VM running [23]. This process of an employed TCB running over the device hardware as a hypervisor structure has various implementation problems in practice, like hardware constraints, system real time behavior, scheduling and access control rights. Apart from TCG’s TPM that offers support for virtualization, solutions based on hardware virtualization (i.e., virtualization assisted through a processor Instruction Set) have been developed for AMD and Intel based systems [26]. In the embedded system domain, though, the type of constraints imposed eliminate the option of using hardware virtualization. The most similar solution for this category of devices is the ARM TrustZone technology [27], which enables the creation of trusted and nontrusted execution environments.

## 4. Proposed Approach for Legacy Systems

While the newest IT based CI devices may have some mechanism of instilling trust, typical CI control devices that constitute the backbone of a CI control loop still have legacy processing units that are not created for security but rather for safety and high, real time, responsiveness. Security Monitoring loggers installed on such devices need to rely on an execution environment that is capable of supporting the ADS sensors’ functionality and that is protected from malicious entities. To achieve high security in legacy devices, it has been proposed in several works to introduce external security tokens that can be considered trusted [23,28,29,30]. Having that in mind, extending the work in [2], we propose a Hardware Security Token (HST) that could be used as an external security element on legacy devices in order to instill a level of trust on collected ADS sensor logs and provide a series of security services to an associated host device and user. In the following subsections we extend, expand and analyze the HST architecture, functionality and services thus structuring a complete solution for CI legacy device security protection.

### 4.1. Hst Architecture

**Figure 1:**
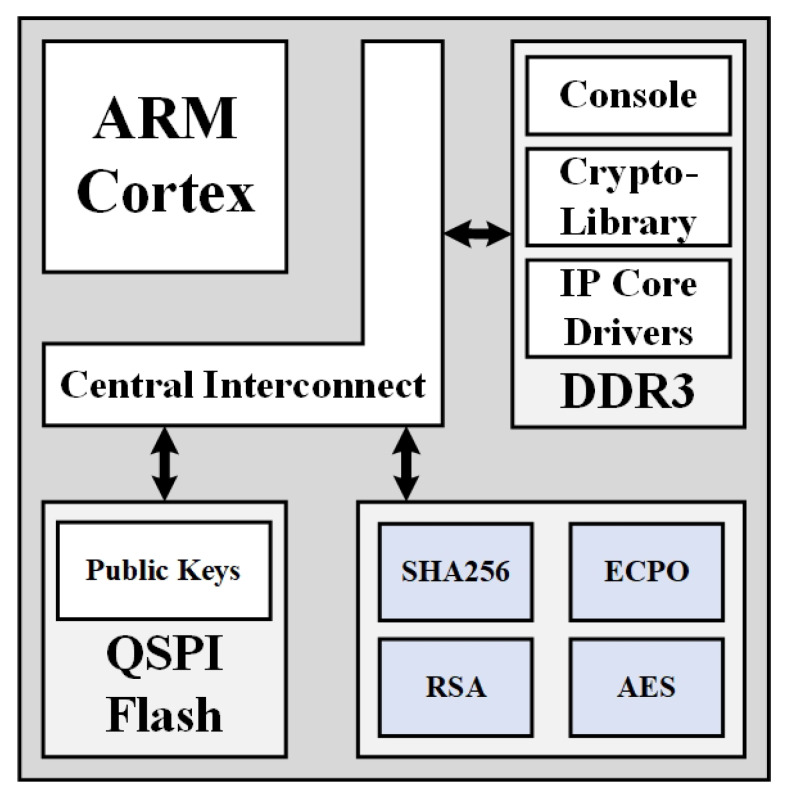
Hardware Security Module Architecture.

The HST is a synchronous System on Chip (SoC) device based on an ARM microprocessor with TrustZone support (e.g., ARM Cortex A processor class) that is connected through an AMBA AXI bus to a series of cryptographic accelerator peripheral IP cores and storage elements like RAM, ROM, and NVRAM memory modules. The cryptography accelerator peripherals act as a security element of the ARM Trustzone enabled processor and consist of an RSA signature unit, an Elliptic Curve (EC) Point Operation unit (ECPO), a SHA256 hash function unit as well as a symmetric key encryption/decryption and key generation unit (using the AES algorithm), following an architecture similar to the one presented in [29]. All the HST IP cores are protected against semi-invasive and non invasive attacks [23,31,32]. The HST, using the above cryptographic peripherals, is capable of generating and verifying digital signatures and certificates, performing key agreement schemes like Elliptic Curve Diffie Hellman Ephemeral (ECDHE) protocol or Needham–Schroeder–Lowe protocol as well as AES based encryption/decryption (AES-CBC, AES-CCM) and authenticated message integrity schemes (HMAC). In addition, the HST has a series of Input/Output Interfaces including CAN bus, USB and Ethernet. The outline of the Hardware-Software hybrid architecture is presented in Figure 1. As can be seen in the above figure, all SoC components are interconnected in the Central Interconnect bus. Apart from hardware IPs, the HST has a software stack that is capable of controlling and coordinating all hardware assisted security operations (using customized Cryptographic IP drivers and cryptography libraries) as well as all communication (through a serial console interface) with an HST host machine. Finally, in the Figure 1, special mention should be made to the non volatile RAM unit which is realized as a QSPI Flash memory. This memory acts as storage space for all cryptographic, sensitive, information like public private keys, symmetric keys, HST states, users, etc.

The AMBA AXI (Central Interconnect) bus provides access to the cryptographic accelerator peripheral IP cores using the software stack. This stack implements a software component that is executed in the ARM cortex A trusted environment. The software component also handles the communication between the HST and the host. During its operation, it polls for an input command given by the host to the HST and collects all the necessary data each specific command requires. The required drivers that enable the stable operation of each IP core are included in this software component. Thus, the input data that have been collected during the command issuance are being propagated to the corresponding cryptographic peripheral for the output result to be calculated. Once this process is completed, our custom Crypto-Library is able to correctly perform a plethora of security protocols and algorithms that inherently depend on the operations our IP Cores provide. Protocols or algorithms that the Crypto-Library features are authenticated message integrity (HMAC), certificate generation and verification, digital signature schemes (ECDSA) as well as many utility operations (key generation, key validation, communication with Flash storage, etc.). The HST outputs to the host through a secure channel the correct output data of the corresponding command.

**Figure 2:**
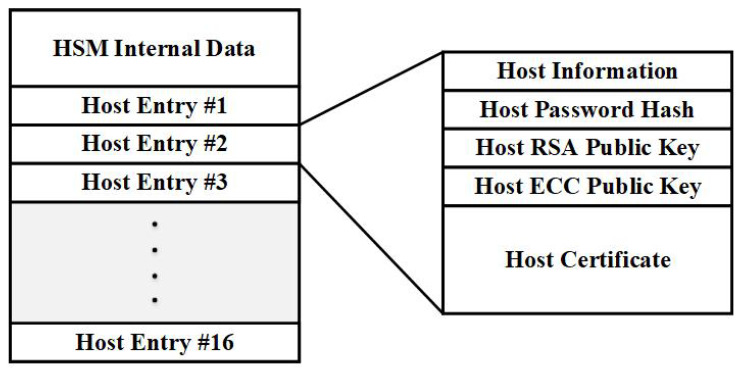
HSM NAND Flash memory contents.

The NVRAM (flash memory) module embedded on the Zynq 7000 series FPGA board can support the validity and functionality of the HST operations in a wide range of various use cases by offering a secure, self-contained and HST controllable storage area where sensitive information can be saved. A typical configuration of an HST’s flash memory contents can be viewed in Figure 2.

First and foremost, stored in the flash memory are all the HST specific information, including the HST ID, status and the highly sensitive private and public RSA and ECC key pairs. The remaining available storage can be utilized in order to store multiple host entries, each containing all the necessary information of the corresponding host. More specifically, a host entry consists of basic host information, including host ID, host type, host status and a copy of the Password Hash that has been generated during the host initialization phase. Additionally, each host’s RSA and ECC public keys are securely stored in the flash memory, along with a certificate that verifies the aforementioned keys (usually an ecdsa-with-sha256 based X-509 certificate due to storage limitations).

Apart from the above mentioned hardware structure, the HST has a dedicated software execution core that is retrieved from the HST flash memory and is loaded in the ARM processor RAM. This software core has dedicated components for the HST communication with the external world. More specifically, the software core enables the HST, extending the functionality described in [2,29], to connect through USB cable to a Host device and through Ethernet to an IP network. The USB serial communication channel serves as a secure means of communication between the Host device and the HST while the Ethernet based IP communication channel serves as a mean of communication between the HST and a remote ADS security monitor and analyzer. Apart from that, the HST software core is responsible for interfacing and usage of the hardware acceleration cryptography IP Cores (for the computationally demanding cryptography operations) using dedicated IP Core drivers. The HST software core also implements lightweight security operations that do not need hardware acceleration as well as security operations that during computations need some dedicated Hardware IP core output. Finally, the HST software core implements the HST API that the HST uses in order to communicate with the associated host device. The HST software component is being used in order to achieve two main operations, the host to HST associated functionality and the HST logging mechanism.

### 4.2. Host to Hst Functionality

The HST can be used in order to identify and authenticate a host device, to collect a series of log entries from the host devices and to transmit them through a secure channel to the ADS monitoring system. In addition, the HST is capable of providing individually, security services to the host devices like certificate generation/verification, digital signatures, key agreement and secure channel establishment. Apart from the actual HST component, the above functionality is manifested through the use of a dedicated software component (HST/host software component) on the host device that acts as a proxy between the Host and the HST. The HST/host software component is operating on an untrusted environment in host device, so it should be assumed that it should not store sensitive information on the host device in a non secure way. This component is also responsible for the authentication of both the host user and the host device to the overall ADS monitoring system. It also generates appropriate log entries using the syslog protocol and secures those entries using the HST. It does not solely rely on the Linux OS syslog mechanism but it also has a dedicated syslog client embedded in its structure in order to minimize a potential attacker’s involvement in the logging approach. Finally, the HST/host software component can rely on the HST commands and messages that are issued by the host user using a dedicated HST Command Line Interface (HST CLI) as well as execute HST CLI scripts.

To achieve a secure use of the HST/host software component and its access to the HST, the approach followed in [2] is adopted and extended. Initially, it can be assumed without lose of generality that before deployment, the HST undergoes an initialization phase. The used and host device that are going to use the HST register their interest in a trusted entity (a trusted host) that uses the registration information (an initial password for the user and a device ID for the host machine) to associate these entities with the HST and the its user. The registration information will be used by the trusted entity to prepare the HST for deployment. More specifically, the HST in its secure processing and storage core will generate a salt (a random value) for the provided password and use both these inputs in a Key Derivation Function (KDF) to create a symmetric cryptography key $Q=Q_{init}$. Apart from the above information, the HST will be used by the Trusted entity in order to generate an Asymmetric cryptography key pair (public and private key) and a associated certificate. This information will act as a secure token and will be encrypted using *Q*. The trusted entity stores in the HST the host/used ID, the salt, the hash outcome of the password and the host’s public key. Finally, the HST generates its own Asymmetric Cryptography public/private key pair. The trusted entity concludes the initialization phase by registering the host to the HST by providing the encrypted host key pair and certificate. The host can store this information in its storage areas (a hard drive disk or flash drive). An attacker that intercepts, copies and analyzes these files will fail to retrieve the key pair since he will not know the password nor the salt. Retrieving this information is very hard for an attacker since the password should not be stored in the host machine nor in the associated HST while the salt is only stored in the HST secure area. Only by a user knowing the password and providing it to the host device connected to the HST associated with this device can he get access to the keys and use the HST services (this acts as a two factor authentication). Failure to provide the appropriate password will generate a log entry (an abnormal event) that will be transmitted through the HST Ethernet dedicated channel to the ADS (using the HST logging mechanism). Taking into account that the HST is considered trusted, the ADS security monitor can trust that the HST sensor’s collected input is not tampered. To achieve that, the entry is digitally signed with the specific HST’s Asymmetric Cryptography key. It can be assumed that the ADS has knowledge of all the HST sensors’ Asymmetric Cryptography public keys and their associated certificates.

When the Initialization phase is finalized, the HST-host system can be deployed in a CI system and provide security services and secure logging. A CI host device and its associated HST are fully connected through USB and the HST services become available when a secure session is established between the pair.

**Figure 3:**
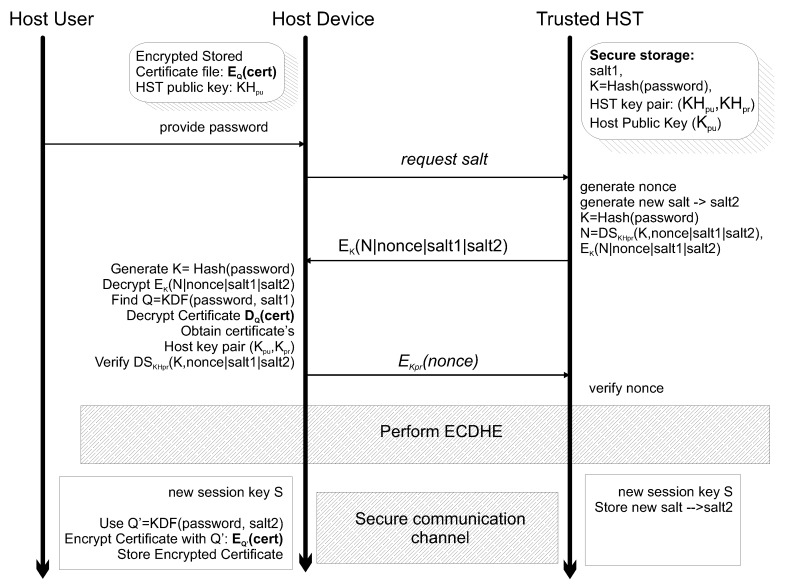
The proposed session key agreement protocol.

The host–HST secure session establishment follows the key agreement scheme proposed in Figure 3. The presented protocol, extending the work in [2], supports a two factor authentication mechanism by combining information of user (user password) and device (the host device secure token). The protocol is built around the Elliptic Curve Diffie Helman Ephemeral (ECDHE) key exchange mechanism for generating a (AES) session key and establishing a secure channel between the host and the HST. The proposed protocol extends the ECDHE by providing a mechanism for securely unlocking during execution the host secure token provided during registration without revealing sensitive information in the process (based on the provided threat/attack model of Section 2.1). Initially, the host user requests to be connected to the HST and thus provides the registered password to the Host device. Note that there is not any form of the password (in clear, encrypted or hashed) stored (apart from the host’s memory) in the host device (e.g., there is no password file). The host device just uses this password to generate its hash function digest. Upon receiving the password by the user (along with the associated username), the HST/host software component installed in the host device sends a request to the HST to receive the HST only stored salt (salt1) in order to decrypt the host secure token (i.e., the encrypted host’s/User’s Certificate and key pair) that is stored in the host disk. The HST then, internally, generates a nonce value that provides replay attack protection, retrieves the hash function digest of the password (that is securely stored) as well as the stored salt (salt1), generates a new salt (salt2) to achieve forward security, concatenates the nonce with salt1 and salt2 and digitally signs the outcome with the HST private key ($KH_{p}r$). Then, the HST concatenates the generated digital signature, the nonce (a random number), the HST stored salt (salt1) and a newly generated salt value (salt2) and using a Symmetric Key encryption algorithm ($E_{K}\left(\right)$: AES) with key the password’s hash function digests. Finally, the HST creates a digital signature $N=DS_{KH_{pr}}\left(K|nonce|salt1|salt2\right)$ and uses it in the encrypted result $E_{K}\left(N|nonce|salt1|salt2\right)$ that eventually sends as a reply to the user’s request. Upon receipt of such message, the host generates his own version of password Hash function digest *K* and tries to decrypt the symmetric key encrypted message $E_{K}\left(N|nonce|salt1|salt2\right)$. Then he can extract the salt (salt1) and through a KDF that uses the password and salt1, recreate the key Q that is necessary to access the stored secure token (user’s certificate and keys). By retrieving the nonce, salt1 and salt2 and by calculating K, the user can verify the digital signature *N* using the HST public key and prove that the HST has knowledge of the password digest *K*. Using the retrieved Host Asymmetric Encryption Keys stored only in memory, the host then sends a digital signature of the nonce to the HST, thus verifying knowledge of both the nonce and his private key ($E_{K_{pr}}$). Then, the ECDHE key agreement scheme is executed using the host retrieved key pair. The outcome of ECDHE is a common session key *S* that can be used for encrypting the remaining traffic between host and HST. The Certificate and Asymmetric cryptography keys are encrypted using the result of a KDF that has as inputs the password along with the new salt (salt2). This result is stored back to the host storage area.

Establishing a secure channel, where traffic is encrypted between the HST and the host, the HST/host software component can forward requests for security services as well as send log messages that will be transmitted with integrity and authenticity through the HST dedicated Ethernet IP communication to the ADS. Integrity and authenticity are achieved by digitally signing the log entries with the retrieved HST Asymmetric Cryptography keys.

### 4.3. Host-Hsm Logging Mechanism

When a security related incident is taking place, it can be detected by the HST/host software component. A log entry is then generated to either be stored using the syslog protocol in the auth.log of the Linux OS as a syslog entry in the host device or to be generated internally in the HST/host software component and forwarded if confidentiality, integrity and authenticity are confirmed to a remote ADS through the HST. Such security incidents can range between possible cryptanalytic attacks, loss of connection between the host and the HST, password authentication failure and many others. The proposed logging mechanism ought to be flexible and simple to use, while containing a sufficient amount of information that can be scaled up according to the application’s needs. Through this scope, each log entry is a JSON message that includes information related to a specific event in the following format (Table 1).

The above structure is always digitally signed with the keys that are stored inside the HST (the host/HST software component does not have access to them). Note that the above log format can be expanded with relative ease to include additional fields of varying type, producing a more detailed log entry that conveys a complete overview of an event with adequate information. The first five basic fields of this JSON array format are necessary for the correct identification of the specific unit that produces a log entry, as well as the exact time the log was generated. The *"HostState"* field provides characterization of the host state relative to the HST. There exist two states in which a CI host can be in, administration and user. In the administrator (*admin*) state, the host user can gain full access to the HSM services and features, including the ability to store other host entries to the HST flash memory, as was analyzed previously. In the *user* state, however, the host user lacks the authorization to add new host entries to the HST. The core of the log entry containing the most important information is the *"event"* array. Its first field, *"type"*, is an integer number indicating the type of event that has been logged. In its current HST realization, the acceptable values of this field are the following types:

- 0: Message integrity validation event.
- 1: Password based host to HST session initiation.
- 2: HST availability.
- 3: Security channel failure.

Directly linked to the above, the field *"failure"* is an integer number that indicates if the occurred event of the type specified in *"type"* has failed (*failure* = 1) or if it has concluded normally (*failure* = 0). The last field encapsulated in the *"event"* is the *"severity"* one, signifying the importance in terms of security impact in cases where event failure is detected. A value of 0 indicates low severity, while the maximum value of 3 marks the logged event with the highest severity. In the final JSON field *"comments"*, additional information related to the logged event can be provided. After generation, the log entry is sent to the ADS monitoring system, containing all the necessary information about the host device, the logged event and its severity. Accordingly, the level of trust and confidence by the ADS is enhanced, providing simultaneously valuable details that aid the appropriate management of the inflicted node or device.

## 5. Hst Practical Conceptualization–Realization

In order to further promote the functionality and applicability of the HST concept and highlight its importance as a flexible and scalable system that provides solutions to many different security problems, CI systems along with trustworthy anomaly detection, in this section we describe a realization of the proposed solution that was implemented during the EU project “CIPSEC:Enhancing Critical Infrastructure Protection with innovative SECurity framework” and that is being expanded in EU projects “CONCORDIA” [33] and “CPSoSaware” [34]. In this realization we specify the HST CLI environment and show its practicality in promoting and accessing the HST security services. Consistency and ease of upgrading should be essential characteristics of this CLI, focusing on scalability and adaptability to a wide range of security scenarios that need to be implemented in multiple CI Systems. These scenarios can vary from simple End-to-End secure channel establishment, to appropriate secure and trusted logging and even to a PKI-like scheme that manages host public keys. In its current realization, the HST is implemented in a Digilent Zedboard device that includes the Zynq 7000 series SoC with ARM Cortex A9 processor and an FPGA fabric on chips (used for the implementation of the HST Hardware IP cores).

### 5.1. Case Study Hst Cli for Cryptographic Application Programming

The availability of a variety of IP cores, as well as the sufficiently powerful Cortex-A9 processing unit offer the ability of implementing numerous cryptographic and security protocols available today. These operations can be accessed directly from the host machine through an in-house built serial CLI. The serial console component accepts commands for execution that adhere to a specific format. Its general structure is as follows: *"command [options] [HostID] [data]"*. For example, in order to execute the Hash-based Message Authentication Code (HMAC) protocol, the host sends to the HST through the serial secure communication channel the command *"hmac [key] [message]"*, where [key] is a secret shared key and [message] the input data of the algorithm. In cases when a command requires extra information related to a specific host like an ECC public key, an extra *[HostID]* field must be added, providing to the HST the ability to extract and use the correct host entry located in its flash memory.

The HST’s hardware board cryptographic features can be accessed through the HST/Host software component. This tool is developed for Linux OS based Host machines and is currently realized for 32 bit and 64 bit x86 Linux platforms as well as ARM Linux platforms. During operation, two different modes are available. In the HST Console mode the various CLI commands are transmitted directly to the HST through a terminal console. In the HST OS mode, on the contrary, all HST commands are given as arguments upon our Linux based HST/host software component executable or as CLI scripts. As it is apparent, OS mode provides greater functionality and flexibility, enabling the development and easy deployment of different applications that want to take advantage of the HST’s features. The overall CLI approach that is being used resembles the openssl library approach with the extension of hardware and dedicated trusted tokens use (hardware in the loop concept, i.e., the HST).

### 5.2. Hst as a Certificate Authority

In a plethora of industrial and CI systems, sensitive information is being exchanged continuously by a wide range of different host machines. This exchange is often exposed to attacks both at a physical and network level, as many hardware sensors and host devices operate in a variety of hostile environments. This is even more prevalent in many Legacy systems that inherently lack strong security design principles. Thus, in such cases the HST functionality can be extended to offer support of certificate management for the different host devices that a CI utilizes, promoting a unique host–HST module as a pseudo Certificate Authority (CA).

Typically, in this scenario, most of the host devices operate in *user* mode, meaning they cannot add another host entry in the HST’s flash memory other than their own entry during the host initialization phase due to the lack of elevated privileges. Each host device has already generated and stored a host ECC private and public key pair. Acting as a pseudo-CA, a host–HST module is operated in *admin* mode. As already mentioned, a host in *admin* state is authorized to store additional information on the flash memory module embedded in its corresponding HST. With the functionality our Crypto-Library offers, the CA can receive Certificate Signing Requests (CSR) from any *user* host operating in the same network as the CA. The CA then checks the validity of the digitally signed CSR and upon successful validation generates an ECC Certificate bound to the specific *user* host. The Certificate is stored on the CA’s corresponding host entry and sent through the network to the host that requested it. Through this process, the CA is in possession of all the user Hosts’ certificates and updates this list whenever a new host is added to the network or a Host regenerates its ECC key pair. Any host from this point forward can request from the CA another host’s certificate, in order to validate the authenticity of its public key and consequentially the ownership of the corresponding private one. Using this PKI-like structure, greater trust is instilled upon the different hosts’ communication than utilizing a basic digital signature scheme without the existence of a CA.

### 5.3. Real-World Test Case Hst Validation

**Figure 4:**
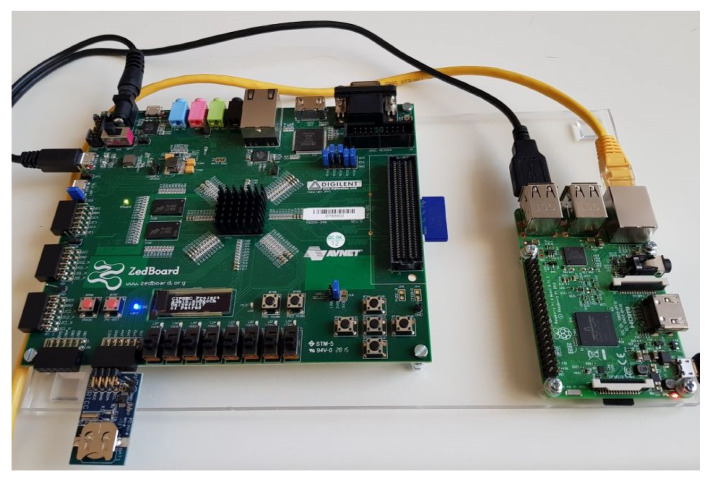
Raspberry Pi and Zedboard host-HSM configuration.

The functionality of the HST has been practically deployed for assessment in two specific CI testbed scenarios. For this purpose, the HST is extended to include a Raspberry Pi module connected to the Zynq 7000 series FPGA board (Zedboard) through a USB serial communication channel. This Pi module, connected to the IP network with either Ethernet or Wi-Fi connection emulates a standalone host machine. In a similar manner, we assume there exist identical host–HST pairs operating across a CI. Such a configuration can be seen in Figure 4.

#### 5.3.1. Test Case A

**Figure 5:**
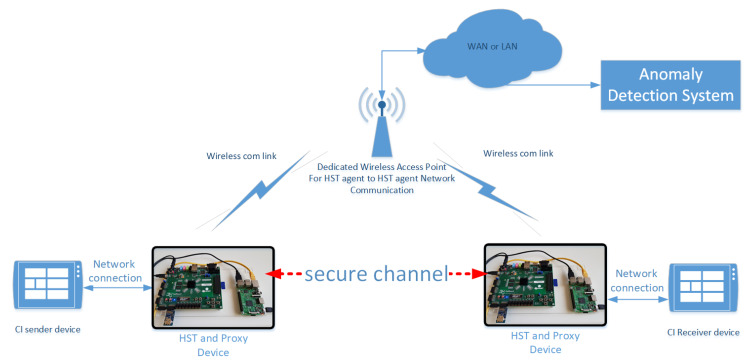
Use Case A: Achieving Confidentiality and Integrity under Man-in-the-Middle (MiTM) and Man-at-the-End (MATE) attack.

The HST can be used to provide End-to-End encryption, integrity and authentication between CI network domains. This functionality in the proposed HST can be related to anomalous behavior since repeated failures in data integrity and authentication constitute a cyberthreat and may signal the beginning of elaborate attack schemes (e.g., advanced Distributed Denial of Service). Having this rationale in mind, the HST concept was adapted accordingly in order to offer the above described functionality. A practical evaluation of the approach was realized in the Deutsche Bahn (DB) NETZE (Germany railways interlocking mechanism provider) infrastructure testing where the host–HST pair was able to capture and redirect UDP packets (used in the RaSTA industrial control protocol used in the DB railway interlocking actuators) destined for another host device in a secure way under a Man-in-the-Middle attack (MitM) and Man-at-the-End (MATE) scenario. The overall use case test configuration is presented in Figure 5. In this mode, the host is exclusively communicating with its associated HST that is responsible for handling the network traffic related to the host machine. The source of this traffic (UDP packets) that is forwarded to the host’s HST, can either be encrypted information from another host–HST pair or data that needs to be encrypted before being forwarded to a destination host–HST pair (in order to achieve End-to-End secure communication). For a mechanism like this to operate properly, the involved HSTs (one for each end point host) execute a key exchange protocol to generate a common shared session key. After this establishment, the raw data a particular host wishes to send to another host must firstly be encrypted and authenticated by the HST (using for example authenticated encryption or encryption and MAC mechanisms) and attached to a UDP packet. The UDP packet is then sent through the network accordingly to the correct destination host–HST pair, where the ciphertext is decrypted and its authenticity–integrity validated, thus revealing the original raw data to the destination host. Utilizing this design philosophy, two hosts can effectively establish a secure channel of communication even over IP, taking advantage of the encryption and decryption services only the HSTs can provide. The logging mechanism on the HST is permanently active in the above mentioned activities in order to detect any failure in the overall process (e.g., wrong key establishment, faulty session establishment, no authentic message, non authentic host user that tries to access the HST, password attacks on the HST or the host, etc.), then the ADS monitoring system is informed through an HST dedicated wireless network channel (instead of an ethernet wired one) due to the testing site policy restrictions. In the validation process of the use case, for integrity HMAC with SHA256 was used, for secure session Establishment ECDHE was used and secure communication was done using AES CCM mode. The exchanged UDP messages as well as the log entry messages to the ADS were digitally signed using ECDSA (using secp256r1 ECC) with SHA256.

**Figure 6:**
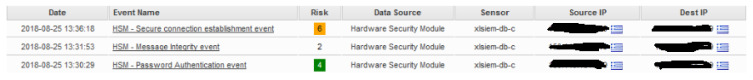
Use Case A: Anomaly Detection and Security Information and Event Management (SIEM) log entries.

**Figure 7:**
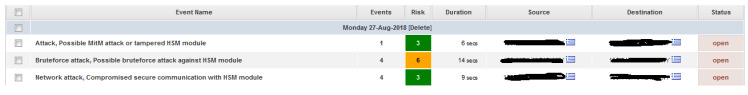
Use Case A: Anomaly Detection and SIEM extracted events.

To validate the above use case and measure the response time of the HST, a MiTM and MATE attack was mounted. In the MiTM attack scenario a malicious user tried to compromise the message integrity and authenticity of the messages during transmission. In the MATE attack scenario, a malicious user tries to bypass the security of the HST, by performing a dictionary attack to find the HST passwords and also maliciously alters log entries of the host device in order to hide its identity. In both the scenarios, all the attacks were captured by the HSTs and the malicious activities were reported to the anomaly detection system. In Figure 6 and Figure 7, the ADS log entries and resulting events are presented.

#### 5.3.2. Test Case B

A very common CI setup consists of a SCADA industrial monitoring system facility that collects information from in-field deployed sensors (e.g., temperature sensors). To emulate the above setup, a test case scenario was created that consists of a server (emulating the SCADA system) and a client (emulating the deployed temperature sensor). Typically, values from the sensor are transmitted through an unprotected channel (e.g., using Modbus protocols) to the SCADA system that is responsible for the fine tuning of the climate conditions in the CI facility. This configuration is prone to Man-in-the-Middle attacks from an adversary that alters the transmitted message and potentially causes severe damage to expensive CI equipment. The emulation process emulates the above scenario using two HST–host configurations as described above and seen in Figure 4 that are connected through a wireless network as a server and a client. One of the HST–host pairs has soldered on the host side (Raspberry Pi) a Pimoroni Enviro pHAT [35], which features a BMP280 temperature/pressure sensor. This sensor is continuously polling for a new temperature value, transmitting it through unprotected UDP packets to the emulated temperature control system. To mount a MitM attack, the Ettercap [36] Open Source tool for Linux was used. Under an unprotected communication channel (without HST), a successful MitM attack was executed, successfully altering the temperature value from its usual range to an extreme one and causing the temperature control system to react accordingly, with unwanted consequences. In order to demonstrate the validity of both the attack and the applied solution, a message integrity mechanism based on hash-based message authentication code (HMAC) is deployed during the communication. The message is hashed and any possible alteration to it is detected by the HST, leading to the rejection of the specific packet. A message integrity failure event is then logged appropriately by the host–HST logging mechanism and transmitted to an operating ADS or SIEM in a similar way as in the Test Case A.

### 5.4. Results and Discussion

Typically, the main issues when it comes to security agents/sensors is that they should be able to respond in time when an attack is taking place or is about to take place, to remain secure under the presence of attacks (hostile environment) and to be able to capture all events associated with a threat or an attack. In this subsection, the measurement results collected during the validation process of the two test cases are presented. It should be noted that the key difference of the HST in terms of response time is the hardware accelerated security primitives that are employed. Thus, the collected measurements are focused on the computation delay for each cryptography primitive operation employed in the test cases that is assisted through hardware means. In Table 2, the time delay accounted from the pointed message is inputted to the HST until it is processed and finally transmitted to the ADS (or a CI end node) is presented for the HST HMAC message integrity and the End-to-End secure communication mechanism (using AES CCM). As it can be observed, the execution time for these operations, due to hardware acceleration, is considerably small if the delays introduced by the communication channel between the HST and the host are also taken into account. When measuring the benefit of hardware implementation versus software ones individually for each security primitive operation inside the HST, as expected, our hardware approach fairs considerably better than purely software designs. For example, using hardware acceleration, an HMAC with SHA256 operation delays 42šec for small byte length inputs versus 62šec when using only software code (67% improvement). Similar improvement appears when the input byte length increases. In addition, for ECDSA digital signature scheme operations, the hardware accelerated solution needed 14.3 ms for signing and 23.7 ms for verification of signatures (using secp256r1 ECCs) versus 24.9 ms and 43.2 ms respectively using only software code (57% improvement in speed).

## 6. Conclusions

In this paper, we propose an approach on how to collect information from CI device ADS sensors that can be trusted and are not tampered with. This approach was based on a hardware assisted dedicated security service provider, the HST, that supports a secure event log and monitoring mechanism. In our approach, the goal is to move securing of security related logs, needed by an ADS, from the Operating System of a CI host (that can be considered insecure) to the HST dedicated hardware module. The HST performs operations in a secure environment and has sole knowledge of cryptography keys that are used for providing confidentiality, integrity and authenticity of the logging mechanism. Thus, even if an attacker manages to compromise the CI host system, he still does not have knowledge of the security keys and also does not have access to the log monitoring mechanism (which in our proposal is fully manifested in the HST). In the paper, we analyzed the proposed approach based on the above described concept and detailed the HST functionality as well as the functionality of the associated HST–host security component deployed in the host device. We also described how the log mechanism could be realized (using JSON data structures) and also provided a practical realization of the HST concept. After describing a manifestation of the HST command line interface, we also described cases study scenarios where the HST can be used to provide even additional services to the secure log monitoring and reporting mechanism.

## Figures and Tables

**Table 1:** HSM Log entry JSON structure.

HSM Log Entry JSON Structure
{
“HostID”:<integer>,
“HostIP”:<integer>,
“HostState”:<string>,
“HSTid”:<integer>,
“timestamp”:<integer>,
“event”:{
“type”:<integer>,
“failure”:<integer>,
“severity”:<integer>
}
“comments”: <string>

**Table 2:** Full execution time of HMAC-SHA256 and AES CCM encryption mechanisms.

Runtime Benchmark				
	**16B**	**64B**	**128B**	**256B**
HMAC	0.049 s	0.061 s	0.071 s	0.092 s
AES	0.052 s	0.067 s	0.089 s	0.135 s
